# Improved Medication Adherence with the Use of Extended-Release Tacrolimus in Liver Transplant Recipients: A Pilot Randomized Controlled Trial

**DOI:** 10.1155/2023/7915781

**Published:** 2023-01-04

**Authors:** Manisha Verma, Radi Zaki, Johnathan Sadeh, John P. Knorr, Mark Gallagher, Afshin Parsikia, Victor Navarro

**Affiliations:** ^1^Department of Medicine, Einstein Healthcare Network, Philadelphia, USA; ^2^Department of Surgery, Einstein Healthcare Network, Philadelphia, USA

## Abstract

**Background:**

Nonadherence to immunosuppression in liver transplant recipients (LTRs) leads to deterioration in health outcomes. Once-dailyextended-release tacrolimus (TAC-ER) may improve adherence when compared to twice-dailyimmediate-release tacrolimus (TAC-IR).

**Methods:**

We conducted a randomized controlled study to evaluate medication adherence, clinical efficacy, and safety of TAC-ER in stable LTR. All patients >18 years who underwent liver transplantation before 6 months were eligible. Patients were randomized 1 : 1 to continued TAC-IR or conversion to TAC-ER. The primary outcome was change in medication adherence from baseline to 9 months, assessed using BAASIS. Secondary outcomes were tacrolimus trough levels, safety, and quality of life.

**Results:**

Thirty-one patients were consented and randomized to either of the two groups: conversion to TAC-ER (*n* = 15) or continued TAC-IR (*n* = 16). Six patients in the TAC-ER group withdrew after randomization due to apprehension about switching medication (*n* = 2), unwillingness to travel (*n* = 2), and increased liver tests after conversion (*n* = 2, both were acute rejections despite therapeutic tacrolimus levels and were considered unrelated to TAC-ER). We compared the results of nine patients in the TAC-ER group that completed the study with those of sixteen in the TAC-IR group. At baseline, there was no difference in tacrolimus trough levels between groups. Improved adherence was observed in the TAC-ER group as 100% of patients reported at least one period of full adherence during the study period (100% vs. 62.6%, *p* = 0.035). Tacrolimus trough levels and liver tests were comparable between groups throughout the study. There were no differences in eGFR, HbA1c, or QoL between the groups.

**Conclusion:**

TAC-ER improved medication adherence while maintaining comparable trough levels, liver function, and QoL as TAC-IR in LTR.

## 1. Introduction

Liver transplant recipients (LTRs) are required to take immunosuppressive medications throughout their life. Nonadherence is common and is variable over time [1]. It is estimated that roughly 20–62% of adults and about 50% of pediatric LTR are nonadherent to their immunosuppressive medication regimen [1, 2]. The most serious outcomes of immunosuppression nonadherence (IMNA) include acute/chronic graft rejection and death. Much of the data pertaining to IMNA derives from the kidney transplant population, but studies have demonstrated worsened graft survival in LTR with IMNA [3]. Meta-analytic data have shown that, on average, 6.7 per 100 LTR per year are nonadherent with immunosuppressant medications [4]. However, this is likely underestimated, with other data reporting IMNA rates as high as 15–40% among LTR [5]. A Scottish article estimated a 30% chronic rejection rate and 10% mortality due to IMNA [6]. In addition to unfavorable patient outcomes, complications related to IMNA contribute to massive costs to the healthcare systems—an estimated $15 to 100 million annually for solid-organ transplants [2].

Nonadherence to medication is not unique to liver transplant patients; it has been seen in patients with kidney, pancreas, heart, and lung transplant as well [7, 8]. Currently, the most common immunosuppressive maintenance protocol prescribed for solid-organ transplants is a triple therapy comprising a calcineurin inhibitor (tacrolimus/cyclosporine), mycophenolate, and a steroid agent [9]. These agents are favored among physicians for their well-understood mechanisms of action and among patients for their relative costs compared to the newer biologic agents. However, when prescribed triple therapy in addition to antimicrobial prophylactic medications, transplant patients face a high pill burden and are required to take multiple tablets daily for the rest of their life [10]. This is likely a significant factor contributing to IMNA, despite patients understanding that these medications are required for their survival [11].

One of the medications in question, tacrolimus, is formulated as either a twice-dailyimmediate-release capsule (TAC-IR, Prograf®) or as a once-dailyextended-release formulation labeled as Envarsus XR® (TAC-ER). Notably, another extended-release formulation (Advagraf/Astagraf) has been commonly used in medication adherence research. Reports in the literature have documented the benefits of the extended-release formulation of tacrolimus, with evidence of increased medication adherence in kidney and kidney-pancreas transplant recipients [12].

We conducted a pilot randomized controlled study to compare the potential benefits and safety of once-daily TAC-ER/Envarsus XR® versus twice-daily TAC-IR in LTR. The primary outcome is a change in self-reported medication adherence from baseline to 3 months. The secondary outcomes include change in the quality of life (QoL) and safety.

## 2. Methods

### 2.1. Study Design

This was a single-center, open-label, two-arm, parallel-group, randomized controlled trial (NCT03386305), enrolling patients from Jan 2018 through June 2021. The study was approved by the local Institutional Review Board (IRB#5024), and written informed consent was obtained from all patients in accordance with the Declaration of Helsinki. Eligible LTR Patients were as follows: (1) ≥ 18 years; (2) at least > 6 months and <5 years post-LT; (3) had stable kidney and liver tests (defined as serum creatinine ≤ 2.5 mg/dL and liver function tests (AST/ALT/alkaline phosphatase (ALP) ≤ 2 times upper limit of normal) for at least 4 weeks before enrollment; (5) on a stable dose of TAC-IR confirmed with maintaining tacrolimus trough levels between 3–12 ng/mL for at least 4 weeks before enrollment. Exclusion criteria were as follows: combined liver-kidney transplantation; concomitant use of a medication with a known cytochrome (CYP) 450 interaction with tacrolimus; received treatment for rejection within 30 days of enrollment; recurrent or active hepatitis C infection or receiving a hepatitis C antiviral agent; and documented gastrointestinal malabsorption. Randomization was conducted using a pregenerated computerized list. Patients were randomized 1 : 1 to continue TAC-IR at their current dose or be converted to TAC-ER (Envarsus XR®; Veloxis Pharmaceuticals, Inc.) at a dose conversion ratio of 0.8 : 1. Subsequent dose adjustments were permitted to maintain tacrolimus trough levels at 4–8 ng/mL, as assessed 7 and 14 days after conversion. Patients converted to TAC-ER were provided the study drug and followed for 9 months, at which time they could decide to continue TAC-ER or return to TAC-IR. Routine adjustment of background immunosuppression (e.g., steroid tapering) was permitted per clinician judgment of transplant surgeons and hepatologists.

### 2.2. Study Outcomes

The primary outcome was change in patient-reported immunosuppression adherence from baseline to 9 months, assessed using the Basel Assessment of Adherence with Immunosuppressive Medications Scale (BAASIS). BAASIS measures taking, skipping, and dose reduction of drugs, with a recall period of 4 weeks. It consists of 4 questions with a 6-point response scale (ranging from never to every day). An additional overall adherence is ranked on a scale of 0 to 100 using a visual analog scale. It can be completed by patients themselves or by an interviewer.

The change in QoL was assessed as a secondary endpoint using PROMIS-29 (Patient-Reported Outcomes Measurement Information System). PROMIS is a result of the NIH's support to develop a psychometrically validated, dynamic system to measure QOL. PROMIS-29 V2.0 comprises a set of 29 questions evaluating the following seven QOL domains: physical function, anxiety, depression, fatigue, sleep disturbance, social function, and pain [13]. The scores are reported as a *T* score for all domains except pain (mean 50, SD = 10) centered on the sample representative of the 2000 US general census considering demographic variables. Pain intensity is assessed using a single item, on a 0–10 scale. It is available in the public domain for research use.

BAASIS and PROMIS-29 were assessed at baseline and at 4 weeks, 3 months, 6 months, and 9 months after the enrollment. Additional secondary efficacy and safety endpoints included tacrolimus trough levels, liver tests (AST, ALT, ALP, GGT), kidney function (serum creatinine, BUN, and eGFR), and hemoglobin A1c collected at baseline and at 3, 6, and 9 months from baseline. The incidence of allograft rejection was collected throughout the study.

### 2.3. Statistical Methods

Given the pilot nature of the study and the complexity of enrolling this population, we aimed to enroll 30 patients in this study. Demographics, safety profile, IMNA, and QoL were compared between the two groups at the baseline and study completion. Adherence to the TAC-IR or TAC-ER is an evolving variable. The overall adherence status was considered by reporting at least one-time full adherence between week 4 and month 9. Continuous variables were examined utilizing the *t*-test or Mann–Whitney *U* test. Chi-squared testing was used for categorical variables. *P* values were 2-sided, and alpha was set at 0.05. All analyses and graphs were performed using the Stata 17 (StataCorp. 2021. Stata Statistical Software: Release 17. College Station, TX: StataCorp LLC.)

## 3. Results

31 patients were consented and randomized. Six patients in the TAC-ER group withdrew after randomization due to apprehension about switching medication while stable (*n* = 2), unwillingness to travel to receive study medications (*n* = 2), and increased liver tests after conversion (*n* = 2, both were deemed acute rejections, despite therapeutic tacrolimus levels but were considered unrelated to TAC-ER conversion). Of these 6, five were male, 4 were African American, and 2 were Caucasian. 9 patients in the TAC-ER group that completed the study, were compared with 16 subjects in the TAC-IR.

### 3.1. Baseline Demographics and Clinical Characteristics

The TAC-ER group was slightly younger but there was no statistical difference (52.0 vs. 55.6 years). The gender predominance was female in the TAC-ER group (78%) and male in the TAC-IR group (62%), but this difference was not statistically significant. Although a nonsignificant difference, the level of education was higher in the TAC-ER group (44% vs. 24%) for a bachelor's degree or above. Time to enrollment was 1.4 years in the TAC-ER group and 1.0 years in the TAC-IR group; enrollment within the first-year after transplant was the same for both groups (44% vs. 47%, *p*=0.92) (Table 1).

The baseline total daily dose was higher in the TAC-ER group (7.2 vs. 4.9 mg, *p*=0.022). Liver tests did not show any statistical difference at baseline or over time. Similarly, kidney function assessed with creatinine, eGFR, and BUN and did not differ significantly at baseline, or through Week 4-Month 9.

### 3.2. Efficacy: Tacrolimus Levels and Rejection

Both the TAC-ER and TAC-IR groups had a comparable mean (±SD) tacrolimus trough levels at the baseline, (7.7 (±2.0) vs. 6.0 (±2.2) ng/mL, *p*=0.096). However, patients in the TAC-ER group were on a higher mean daily dose of tacrolimus at the baseline (7.2 (±1.8) vs. 4.9 (±2.6) mg, *p*=0.022).

**Figure 1:**
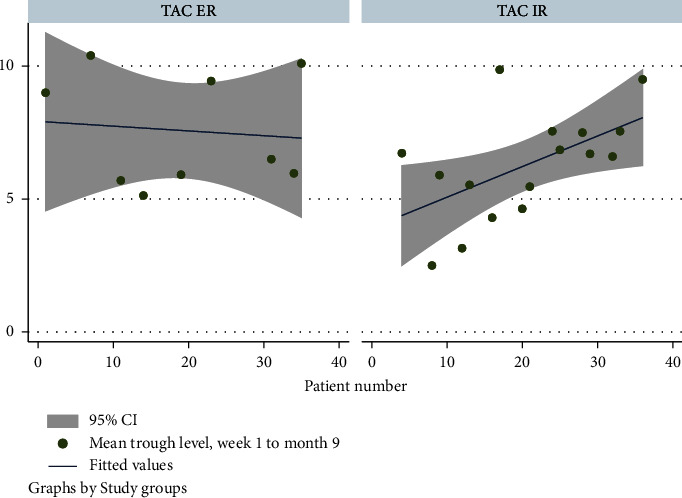
Mean PROMIS-29 scores, week 4-month 9.

Mean tacrolimus trough levels from week 1 through month 9 were comparable between TAC-ER and TAC-IR groups (7.6 (±2.1) vs. 6.3 (±2.0) ng/mL, *p*=0.14) (Figure 1). There was one episode of acute rejection noted in each group (*p*=0.76).

### 3.3. Medication Adherence

Using the BAASIS instrument, we considered three levels of adherence: never taken as prescribed, sometimes taken as prescribed, and always taken as prescribed.

At baseline, fewer patients in the TAC-ER group noted IMNA within the four weeks prior to enrollment; however, this was not statistically significant (33% vs. 62%, *p*=0.16). During the study period, the pattern of adherence changed at 4 weeks, 3 months, 6 months, and 9 months. Full adherence (always taken as prescribed) throughout the entire study was only reported in three patients, two in the TAC-ER group and one in the TAC-IR group; however, this was not statistically significant. The proportion of patients who reported at least one-time full adherence over the study period was greater in the TAC-ER vs. the TAC-IR group (100% vs. 62%, *p*=0.035). Notably, the percentage of patients reporting better adherence remained higher in the study group than in the control over time.

### 3.4. Quality of Life (Table 2 and Figure 2)

At the baseline, physical function and social function in our study population were below the mean (SD) 50 (10) of the general population. No component of PROMIS-29 differed between groups. However, the mean score for sleep disturbances trended towards better sleep in the TAC-ER group (54.3 (±4.2) vs. 51.2 (±3.6), *p*=0.069). No significant change in other PROMIS domain scores was found over time.

## 4. Discussion

Nonadherence has been identified as a major modifiable risk factor for poor outcomes in liver transplantation by the Consensus on Managing Modifiable Risk in Transplantation (COMMIT) group [14]. Studies have shown that IMNA is often a persistent problem and one which may actually worsen with time [15, 16]. Minimizing pill burden and using once-daily dosing is a potential strategy to address nonadherence [17, 18]. We report better medication adherence with the use of once-daily TAC-ER when compared to twice-daily TAC-IR. There were no differences in clinical or safety outcomes between the two study groups.

Clinical data using TAC-ER in the setting of liver transplantation are limited. A phase 2 study published by Alloway et al. demonstrated safe conversion to TAC-ER in 57 stable LTR who were a median of 32.2 months after transplant [19]. Of these, 43 subjects completed 52 weeks of treatment in an extension phase of the study. The mean therapeutic dose was 30% lower with TAC-ER after conversion (6.1 mg and 4.8 mg). There were three discontinuations due to adverse events in the TAC-ER group, and one possibly related rejection in the TAC-ER group which was resolved. In a pharmacokinetic phase 2 study, 58 LTR were randomized to de novo TAC-ER or TAC-IR, with 35 subjects completing a 52-week extension period [20]. Adverse event rates were similar between groups, and there were 6 and 4 rejections observed in the TAC-ER and TAC-IR groups, respectively. Our study did not show significant differences in adverse events or rejections in a 9-month study period after conversion.

Adherence is unstable and varies over time. Longer-term studies with TAC-ER would be of great interest, especially considering a recent European Liver Transplant Registry study of over 13,000 patients, which demonstrated superior long-term patient and graft survival (up to 8 years) in LTR either initiated and maintained on, or converted to, TAC-ER [21]. Our study has notable limitations, particularly high drop-out rates, low sample size, and imbalance in a number of patients in the two comparative arms. There were patient-related factors such as unwillingness to take the risk of converting to a new medication when they were already on a stable dose and difficulty in coming to the clinic for research visits and picking up study medications (while their current medications could be shipped directly to their home). Moreover, the lack of blinding may have added bias to our patient's responses, though admittedly it is difficult to blind patients when the number of pills taken during the day is by nature impossible to hide from the patient.

In conclusion, this study suggests improved adherence and acceptable clinical outcomes after conversion to TAC-ER in a stable liver transplant population. A larger and longer-term study is needed to assess the impact of improved adherence on clinical outcomes after conversion to TAC-ER.

## Figures and Tables

**Figure 2:**
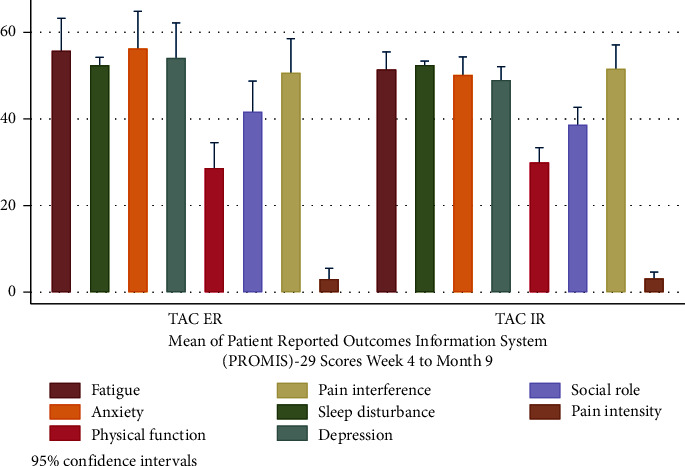
Mean trough levels in the TAC ER and TAC IR groups (week 1 through month 9).

**Table 1:** Baseline demographics, clinical characteristics, and medication adherence of the study participants.

	TAC-ER (*n* = 9)	TAC-IR (*n* = 16)	*p* value
Age, mean (SD)	52 (12.5)	55.6 (12.2)	0.49
Ethnicity, *n* (%)			
Caucasian	6 (67%)	10 (62%)	0.86
African American	2 (22%)	4 (25%)	
Hispanic	1 (11%)	1 (6%)	
Asian	0 (0%)	1 (6%)	
Gender, *n* (%)			
Male	2 (22%)	10 (62%)	0.053
Female	7 (78%)	6 (38%)	
Level of education, *n* (%)			
High school/GED	5 (56%)	6 (38%)	0.21
Some college	0 (0%)	6 (38%)	
Bachelors	3 (33%)	3 (19%)	
Masters or above	1 (11%)	1 (6%)	
Marital status, *n* (%)			
Single	4 (44%)	3 (19%)	0.31
Married	5 (56%)	9 (56%)	
Divorced	0 (0%)	3 (19%)	
Widowed	0 (0%)	1 (6%)	
Time from LT to enrollment, median years (IQR)	1.4 (0.8, 2.8)	1.0 (0.6, 3.0)	0.82
Time from LT to enrollment <1 yr, *n* (%)	4 (44%)	7 (47%)	0.92
Baseline tacrolimus trough level, mean ng/mL (SD)	7.7 (2.0)	6.0 (2.2)	0.096
Baseline tacrolimus dose, mean mg/day (SD)	7.2 (1.8)	4.9 (2.6)	0.022
Mean trough level ng/mL, week 1-month 9 (IQR)	6.5 (9.4)	6.65 (7.5)	0.28
Mean trough level ng/mL, week 1-month 9, mean (SD)	7.6 (2.1)	6.3 (2.0)	0.14
Acute rejection, *n* (%)	1 (17%)	1 (11%)	0.76
Any nonadherence at baseline	3 (33%)	10 (62%)	0.16
BAASIS adherence at baseline			
Never taken as prescribed	0 (0%)	1 (6%)	0.33
Sometimes taken as prescribed	3 (33%)	9 (56%)	
Always taken as prescribed	6 (67%)	6 (38%)	
BAASIS adherence at 4 weeks			
Never taken as prescribed	0 (0%)	0 (0%)	0.22
Sometimes taken as prescribed	2 (29%)	8 (57%)	
Always taken as prescribed	5 (71%)	6 (43%)	
BAASIS adherence at 3 months			
Never taken as prescribed	0 (0%)	1 (8%)	0.053
Sometimes taken as prescribed	2 (22%)	8 (67%)	
Always taken as prescribed	7 (78%)	3 (25%)	
BAASIS adherence at 6 months			
Never taken as prescribed	0 (0%)	0 (0%)	0.26
Sometimes taken as prescribed	3 (38%)	7 (64%)	
Always taken as prescribed	5 (62%)	4 (36%)	
BAASIS adherence at 9 months			
Never taken as prescribed	0 (0%)	0 (0%)	0.14
Sometimes taken as prescribed	1 (14%)	7 (47%)	
Always taken as prescribed	6 (86%)	8 (53%)	
Full-adherence throughout the study	2 (22%)	1 (6%)	0.24
At least one-time full adherence	9 (100%)	10 (62%)	0.035

**Table 2:** PROMIS-29 scores, baseline, and mean throughout the study.

		TAC-ER (*n* = 9)	TAC-IR (*n* = 16)	*p* value
Fatigue, mean (SD)	Baseline	51.1 (14.2)	47.4 (9.1)	0.44
	Week 4-month 9	55.7 (9.7)	51.4 (7.7)	0.23

Sleep disturbance, mean (SD)	Baseline	54.3 (4.2)	51.2 (3.6)	0.069
	Week 4-month 9	52.4 (2.4)	52.4 (1.7)	0.99

Anxiety, mean (SD)	Baseline	52.2 (12.1)	47.3 (7.2)	0.23
	Week 4-month 9	56.2 (11.2)	50.1 (7.8)	0.12

Depression, mean (SD)	Baseline	50.4 (10.3)	45.6 (5.8)	0.16
	Week 4-month 9	54.1 (10.5)	48.9 (5.9)	0.13

Physical function, mean (SD)	Baseline	29.1 (7.0)	28.3 (5.6)	0.77
	Week 4-month 9	28.6 (8.0)	29.9 (6.6)	0.67

Social roles, mean (SD)	Baseline	41.4 (9.2)	27.0 (8.3)	0.24
	Week 4-month 9	41.7 (9.2)	38.6 (7.5)	0.38

Pain interference, mean (SD)	Baseline	52.9 (11.8)	51.9 (10.7)	0.84
	Week 4-month 9	50.6 (10.3)	51.5 (10.4)	0.83

Pain intensity, mean (SD)	Baseline	3.5 (3.9)	2.9 (3.9)	0.70
	Week 4-month 9	3.1 (3.2)	3.2 (2.8)	0.92

## Data Availability

Data are available from the corresponding author upon request.

## Abbreviations

LTR: Liver transplant recipients
BAASIS: Basel assessment of adherence with immunosuppressive medications scale
IMNA: Immunosuppression nonadherence
TAC-ER: Extended-release tacrolimus
TAC-IR: Immediate-release tacrolimus.

## References

[1] Whitsett M., Levitsky J. (2018). Medication nonadherence in liver transplantation. *Clinical Liver Disease*.

[2] Wadhwani S. I., Nichols M., Klosterkemper J. (2020). Implementing a process to systematically identify and address poor medication adherence in pediatric liver transplant recipients. *Pediatric Quality & Safety*.

[3] Lieber S. R., Volk M. L. (2013). Non-adherence and graft failure in adult liver transplant recipients. *Digestive Diseases and Sciences*.

[4] Dew M. A., DiMartini A. F., De Vito Dabbs A. (2007). Rates and risk factors for nonadherence to the medical regimen after adult solid organ transplantation. *Transplantation*.

[5] Burra P., Germani G., Gnoato F. (2011). Adherence in liver transplant recipients. *Liver Transplantation*.

[6] O’Carroll R. E., McGregor L. M., Swanson V., Masterton G., Hayes P. C. (2006). Adherence to medication after liver transplantation in Scotland: a pilot study. *Liver Transplantation*.

[7] Kung M., Koschwanez H. E., Painter L., Honeyman V., Broadbent E. (2012). Immunosuppressant nonadherence in heart, liver, and lung transplant patients: associations with medication beliefs and illness perceptions. *Transplantation*.

[8] Castleberry A. W., Bishawi M., Worni M. (2017). Medication nonadherence after lung transplantation in adult recipients. *The Annals of Thoracic Surgery*.

[9] Kwong A. J., Kim W. R., Lake J. R. (2021). OPTN/SRTR 2019 annual data report: liver. *American Journal of Transplantation*.

[10] Serper M., Patzer R. E., Reese P. P. (2015). Medication misuse, nonadherence, and clinical outcomes among liver transplant recipients. *Liver Transplantation*.

[11] Paterson T. S. E., Demian M., Shapiro R. J., Loken Thornton W. (2019). Impact of once- versus twice-daily tacrolimus dosing on medication adherence in stable renal transplant recipients: a Canadian single-center randomized controlled trial. *Canadian Journal of Kidney Health and Disease*.

[12] Torabi J., Campbell A., Ajaimy M., Rocca J. P., Graham J. A. (2018). Utilization of LCP-tacrolimus (Envarsus XR) in simultaneous pancreas and kidney transplant recipients. *The Ochsner Journal*.

[13] Cella D., Yount S., Rothrock N. (2007). The Patient-Reported Outcomes Measurement Information System (PROMIS): progress of an NIH Roadmap cooperative group during its first two years. *Medical Care*.

[14] Neuberger J. M., Bechstein W. O., Kuypers D. R. J. (2017). Practical recommendations for long-term management of modifiable risks in kidney and liver transplant recipients: a guidance report and clinical checklist by the consensus on managing modifiable risk in transplantation (COMMIT) group. *Transplantation*.

[15] Lieber S. R., Helcer J., Leven E. (2018). Pretransplant psychosocial risk factors may not predict late nonadherence and graft rejection in adult liver transplant recipients. *Exp Clin Transplant*.

[16] Fine R. N., Becker Y., De Geest S. (2008). Nonadherence consensus conference summary report. *American Journal of Transplantation*.

[17] Ettenger R., Albrecht R., Alloway R. (2018). Meeting report: FDA public meeting on patient-focused drug development and medication adherence in solid organ transplant patients. *American Journal of Transplantation*.

[18] Doyle I. C., Maldonado A. Q., Heldenbrand S., Tichy E. M., Trofe-Clark J. (2016). Nonadherence to therapy after adult solid organ transplantation: a focus on risks and mitigation strategies. *American Journal of Health-System Pharmacy*.

[19] Alloway R. R., Eckhoff D. E., Washburn W. K., Teperman L. W. (2014). Conversion from twice daily tacrolimus capsules to once daily extended-release tacrolimus (LCP-Tacro): phase 2 trial of stable liver transplant recipients. *Liver Transplantation*.

[20] DuBay D. A., Teperman L., Ueda K. (2019). Pharmacokinetics of once-dailyextended-release tacrolimus tablets versus twice-daily capsules in de novo liver transplant. *Clinical Pharmacology in Drug Development*.

[21] Adam R., Karam V., Cailliez V. (2019). Improved survival in liver transplant patients receiving prolonged-releasetacrolimus-based immunosuppression in the European liver transplant Registry (ELTR): an extension study. *Transplantation*.

